# Imaging Diverse Pathogenic Bacteria In Vivo with ^18^F-Fluoromannitol PET

**DOI:** 10.2967/jnumed.122.264854

**Published:** 2023-05

**Authors:** Spenser R. Simpson, Alexandria E. Kesterson, Justin H. Wilde, Zoraiz Qureshi, Bijoy Kundu, Mark P. Simons, Kiel D. Neumann

**Affiliations:** 1 Department of Diagnostic Imaging, St. Jude Children’s Research Hospital, Memphis, Tennessee;; 2 Department of Radiology and Medical Imaging, University of Virginia, Charlottesville, Virginia;; 3 Combat Wounds Division, Naval Medical Research Center, U.S. Navy, Silver Spring, Maryland;; 4 Department of Computer Science, University of Virginia, Charlottesville, Virginia;; 5 Department of Biomedical Engineering, University of Virginia, Charlottesville, Virginia; and; 6 Department of Chemistry, University of Virginia, Charlottesville, Virginia

**Keywords:** ^18^F, PET imaging, infection, bacteria

## Abstract

Infectious disease remains the main cause of morbidity and mortality throughout the world. Of growing concern is the rising incidence of multidrug-resistant bacteria, derived from various selection pressures. Many of these bacterial infections are hospital-acquired and have prompted the Centers for Disease Control and Prevention in 2019 to reclassify several pathogens as urgent threats, its most perilous assignment. Consequently, there is an urgent need to improve the clinical management of bacterial infection via new methods to specifically identify bacteria and monitor antibiotic efficacy in vivo. In this work, we developed a novel radiopharmaceutical, 2-^18^F-fluoro-2-deoxy-mannitol (^18^F-fluoromannitol), which we found to specifically accumulate in both gram-positive and gram-negative bacteria but not in mammalian cells in vitro or in vivo. **Methods:** Clinical isolates of bacteria were serially obtained from wounds of combat service members for all in vitro and in vivo studies. Bacterial infection was quantified in vivo using PET/CT, and infected tissue was excised to confirm radioactivity counts ex vivo. We used these same tissues to confirm the presence of bacteria by extracting and correlating radioactive counts with colony-forming units of bacteria. **Results:** ^18^F-fluoromannitol was able to differentiate sterile inflammation from *Staphylococcus aureus* and *Escherichia coli* infections in vivo in a murine myositis model using PET imaging. Our study was extended to a laceration wound model infected with *Acinetobacter baumannii,* an important pathogen in the nosocomial and battlefield setting. ^18^F-fluoromannitol PET rapidly and specifically detected infections caused by *A. baumannii* and several other important pathogens (*Enterococcus faecium, S. aureus, Klebsiella pneumoniae, A. baumannii, Pseudomonas aeruginosa,* and *Enterobacter* spp.). Importantly, ^18^F-fluoromannitol PET was able to monitor the therapeutic efficacy of vancomycin against *S. aureus* in vivo. **Conclusion:** The ease of production of ^18^F-fluoromannitol is anticipated to facilitate wide radiopharmaceutical dissemination. Furthermore, the broad sensitivity of ^18^F-fluoromannitol for bacterial infection in vivo suggests that it is an ideal imaging agent for clinical translation to detect and monitor infections and warrants further studies in the clinical setting.

## Visual Abstract

**Figure:**
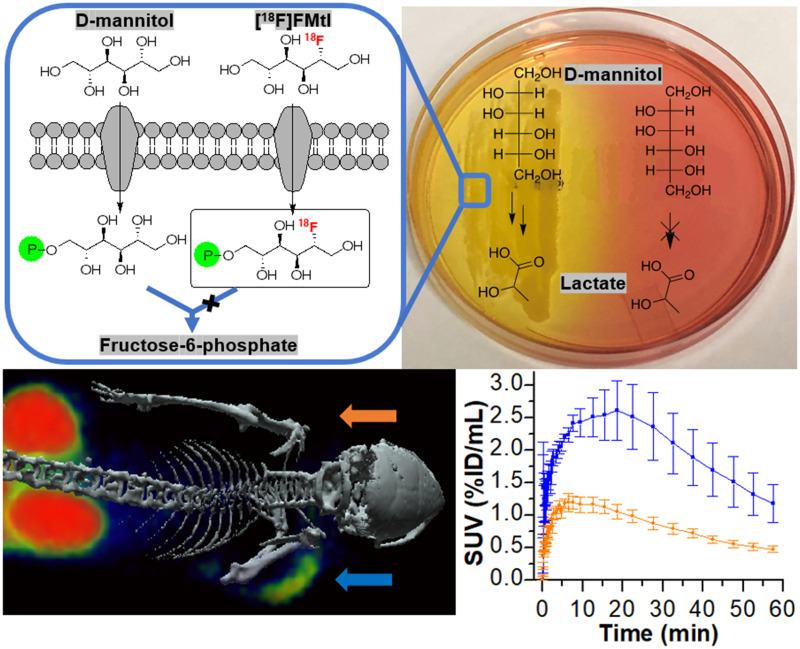

Infection is responsible for the highest morbidity and the third most deaths among all human diseases worldwide (1). Most health-care–associated infections in the United States arise from several common pathogens, including *Staphylococcus aureus, Acinetobacter baumannii, Pseudomonas aeruginosa,* and those of the Enterobacteriaceae family (*Escherichia coli* and *Salmonella* spp., among others). The rising trend of antimicrobial resistance, compounded by a growing population of immunocompromised individuals (HIV/AIDS, chemotherapy, organ transplantation, diabetes) creates an enormous economic strain on the U.S. health-care system, with estimates ranging from $28 billion to $45 billion annually (2). Current estimates project that drug-resistant infections will become the leading cause of global death, surpassing cancer-associated mortality by 2050 (3). The Centers for Disease Control and Prevention has recently listed carbapenem-resistant *Acinetobacter* and Enterobacteriaceae, extended-spectrum β-lactamase–producing Enterobacteriaceae, multidrug-resistant *P. aeruginosa,* methicillin-resistant *S. aureus,* and others as urgent or serious threats to human health (4). Carbapenem-resistant *A. baumannii* alone was responsible for 8,500 hospitalizations, 700 deaths, and $281 million in U.S. health-care costs in 2017 (4). Carbapenem-resistant *A. baumannii* infections are particularly problematic for patients who have comorbidities or are immunocompromised (5); however, *A. baumannii*–associated infections are also well-described complications of severe combat-related injuries in military service members (6). Accordingly, there is an urgent need to improve the diagnosis and treatment of bacterial infection.

Traditional approaches to diagnosing infection include obtaining a biopsy sample from tissue or blood and subsequently culturing pathogens in media to identify an organism. Bacterial cultures from tissue biopsy specimens remain the gold standard for confirming the presence, identity, and drug sensitivity of a microorganism; however, deep-seated infections that are difficult to access or identify often rely on noninvasive imaging techniques based on changes in anatomy or tissue morphology. The most common anatomic imaging modalities used, such as CT and MRI, are frequently nonspecific for delineating active infection from sterile inflammatory disease. Nuclear medicine uses labeled leukocytes (^99m^Tc- or ^111^In-oxine) (7) and ^67^Ga-citrate scintigraphy (8), which rely on indirect measurements of leukocyte recruitment to an area of interest. PET imaging with ^18^F-FDG is increasingly used; however, none of these imaging techniques can distinguish active infection from cancer or inflammation. Consequently, current clinically available imaging techniques are not adequately specific to diagnose deep-seated infection.

To address this challenge, many recently developed radiopharmaceuticals seek to exploit various bacteria-specific signatures such as metabolism (9–11), cofactor biosynthesis (12*,*^13^), and labeled antibiotics (14*,*^15^). Despite these scientific advances, a dire need persists for imaging agents that meet the challenges of clinical infectious disease practice; the ideal agent should possess broad bacterial strain sensitivity, have optimal pharmacokinetics (rapid target engagement, clearance of nonspecific signals to promote contrast), and be widely deployable and available for clinical use.

The phosphoenolpyruvate-dependent sugar phosphotransferase system catalyzes phosphorylation of incoming sugar substrates, with concomitant translocation across the cell membrane, and is widely found in bacteria (16–18). Because of this metabolic signature, ^3^H- and ^14^C-d-mannitol analogs were recently evaluated in a panel of pathogens (19). We hypothesized that a positron-emitting analog of mannitol, 2-^18^F-fluoro-2-deoxy-mannitol (^18^F-fluoromannitol), would be a specific precursor for bacterial metabolism and, subsequently, a suitable imaging agent for in vivo use with PET. Here, we report a simple, widely deployable radiosynthesis of ^18^F-fluoromannitol and demonstrate that this imaging agent possesses broad-spectrum bacterial sensitivity both in vitro and in vivo using clinical isolates of bacteria from combat wounds in military service members. Moreover, we demonstrate that ^18^F-fluoromannitol can quantify antimicrobial efficacy in vivo.

## MATERIALS AND METHODS

### Manual Radiosynthesis of ^18^F-Fluoromannitol

^18^F-fluoromannitol was synthesized from commercially available cyclotron-derived ^18^F-fluoride ions and isolated in a radiochemical yield of 23% ± 2% (end of synthesis) with an estimated molar activity of 5.5 ± 0.37 GBq/μmol (*n* = 14). Detailed radiosynthetic procedures are described in the supplemental methods (supplemental materials are available at http://jnm.snmjournals.org).

### Murine Myositis Model

CBA/J mice (male, 5–6 wk old) were inoculated with 50 μL (typical inoculations were 10^6^ colony forming units [CFUs]) of bacteria into the triceps brachii muscle as previously described (10*,*^12^*,*^20^).

### Wound Infection Model

C57BL/6 mice (male, 5–6 wk old) were used for all experiments. A 3-mm laceration in the dorsal fascia was injected with 50 μL (typical inoculations were 10^6^ CFUs) of *A. baumannii* into the open wound, and the infection was allowed to develop for 6 h (11) before imaging.

### PET/CT Imaging

For all studies, 5.5 ± 1.8 MBq were injected via a lateral tail-vein catheter. After injection, mice were imaged by dynamic (60 min) or static (45–60 min) PET acquisition. All scans were immediately followed by a 10-min CT scan for attenuation correction and anatomic coregistration. Afterward, the mice were euthanized for biodistribution studies and CFU analysis when applicable. γ-counting of harvested tissue was performed using an automatic γ-counter (Hidex). Detailed protocols are described in the supplemental methods.

### Computation, Registration, and Quantification of Parametric PET Maps

Parametric PET maps of the total rodent body were generated and computed as previously described (21). Net influx rate maps were computed and coregistered with CT images using PMOD (version 3.9, PMOD Technologies). The regional average net influx rate was quantified and correlated with CFUs. Detailed protocols are described in the supplemental methods.

### Statistical Methods

Quantitative data are expressed as mean ± SEM unless otherwise noted. Means were compared using 1-way ANOVA or, for multiple comparisons, 2-way ANOVA. The Mann–Whitney *U* test was used to test significant differences in SUV comparisons over time (dynamic imaging). *P* values smaller than 0.05 were considered statistically significant.

## RESULTS

### ^18^F-Fluoromannitol Radiosynthesis

**FIGURE 1.**
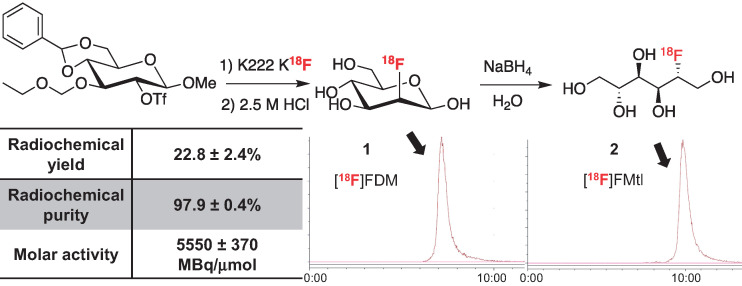
Radiochemical synthesis of ^18^F-fluoromannitol is a straightforward 3-step, 2-pot process using commercially available precursor. Radiosynthesis produces ^18^F-fluoromannitol in high radiochemical yield and purity, which are easily determined by radio–high-performance liquid chromatography. Molar activity was measure of 3 radiosyntheses. Intermediates were verified using fully characterized ^19^F isotopic standard and matched to high-performance liquid chromatography retention times. FDM = 2-^18^F-fluoro-2-deoxy-mannose; FMtl = fluoromannitol.

The radiosynthesis of ^18^F-fluoromannitol commences through a 2-step, 1-pot production of 2-^18^F-fluoro-2-deoxy-mannose (**1**) (Fig. 1). We synthesized the fully protected ^19^F-isotopic precursor of 2-^18^F-fluoro-2-deoxy-mannose (**9**) (Supplemental Schemes 1–3) to identity the ^18^F-labeled intermediate (Supplemental Figs. 1 and 2) and calculate the molar activity (Supplemental Figs. 3–5) by high-performance liquid chromatography. The production of ^18^F-fluoromannitol generates a 7.31 ± 0.25 μg/mL concentration of ^19^F-fluoromannitol, which meets the Food and Drug Administration microdose definition (22) and is suitable for clinical studies. 2-^18^F-fluoro-2-deoxy-mannose is converted to ^18^F-fluoromannitol (**2**) (Fig. 1) by sodium borohydride–mediated reduction and isolated in more than 99% radiochemical purity in a 23% ± 2% radiochemical yield (*n* = 14) (end of synthesis) and an estimated molar activity of 5.5 ± 0.4 GBq/μmol.

### Characterization of ^18^F-Fluoromannitol In Vitro

**FIGURE 2.**
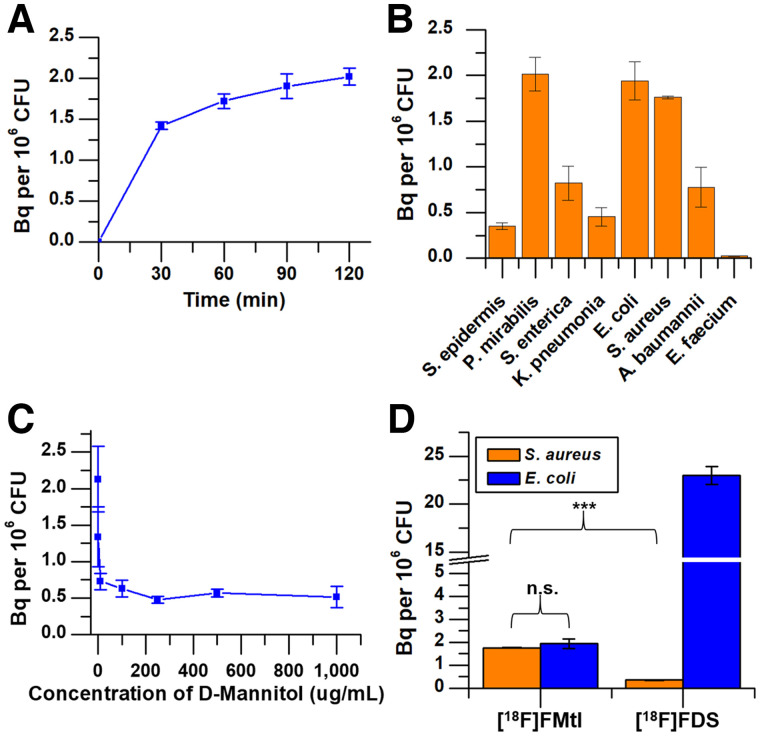
Accumulation of ^18^F-fluoromannitol in bacteria in vitro. (A) Rapid accumulation of ^18^F-fluoromannitol in *S. aureus* cultures, commensurate with ideal imaging times. (B) Uptake of ^18^F-fluoromannitol in pathogens of clinical interest determined at 60 min of incubation. (C) Competitive uptake assay of ^18^F-fluoromannitol in presence of unlabeled d-mannitol in *S. aureus*. (D) *S. aureus* and *E. coli* cultures incubated with ^18^F-fluoromannitol or ^18^F-FDS. Data are mean ± SEM (*n* = 6). ***P < 0.001. FMtl = fluoromannitol; n.s. = not statistically significant.

Over time, both *S. aureus* and *E. coli* readily incorporated ^18^F-fluoromannitol (Fig. 2A) but not heat-killed bacteria, demonstrating metabolic specificity of bacteria for ^18^F-fluoromannitol. We next evaluated accumulation of ^18^F-fluoromannitol in a broad panel of bacterial strains (Supplemental Table 1). All strains tested, except for *P. aeruginosa* and *Enterococcus faecium,* showed rapid and significant accumulation (Fig. 2B). Coincubation of ^18^F-fluoromannitol with d-mannitol in *S. aureus* cultures demonstrated target specificity and that accumulation of ^18^F-fluoromannitol is not concentration-dependent (Fig. 2C); concentrations of at least 10 μg/mL of d-mannitol blocked ^18^F-fluoromannitol accumulation in bacteria. We also compared the accumulation of ^18^F-fluoromannitol in *S. aureus* (gram-positive) and *E. coli* (gram-negative) cultures against ^18^F-FDG, the current workhorse of nuclear medicine, and 2-deoxy-2-^18^F-fluorosorbitol (^18^F-FDS), which has demonstrated high specificity for Enterobacteriaceae organisms (10). As anticipated, ^18^F-fluoromannitol accumulated in both *E. coli* and *S. aureus,* and in *S. aureus* this accumulation was significantly higher than that of ^18^F-FDS (*P* < 0.001) (Fig. 2D; Supplemental Fig. 6). The accumulation of ^18^F-fluoromannitol in *S. aureus* and *E. coli* did not significantly differ (*P* = 0.64), and the accumulation of ^18^F-FDS in *S. aureus* did not significantly differ from that in negative control (10-times heat-killed bacteria, *P* = 0.35; Supplemental Fig. 7). Taken together, these data show that ^18^F-fluoromannitol accumulates rapidly in a wide panel of bacteria and thus may serve as a broad-spectrum imaging agent of infection in vivo.

### ^18^F-Fluoromannitol Characterization In Vivo

**FIGURE 3.**
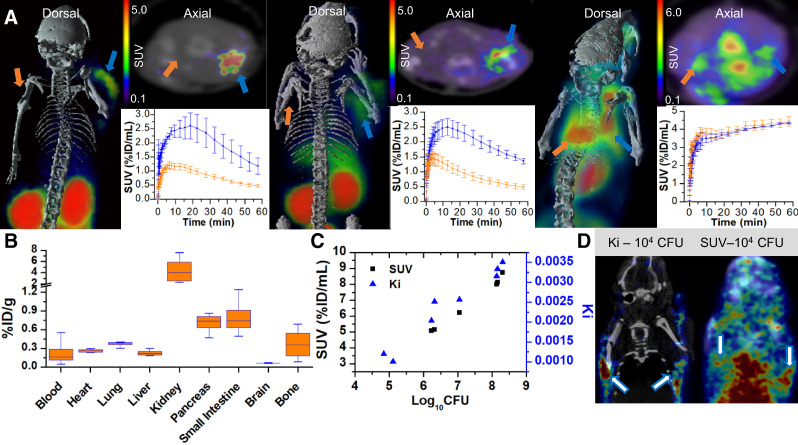
In vivo quantification of bacterial infection by ^18^F-fluoromannitol imaging. (A) ^18^F-fluoromannitol signal is significantly elevated in infected triceps brachii (blue arrows) but not in inflamed triceps brachii (orange arrows) in both *S. aureus* (left) and *E. coli* (middle); ^18^F-FDG cannot differentiate infection from sterile inflammation (right) (4 each; *P* < 0.001). (B) ^18^F-fluoromannitol ex vivo biodistribution was performed on indicated tissues of interest after imaging. Data are mean with interquartile range (*n* = 4). (C) ^18^F-fluoromannitol imaging (SUV or net influx rate) was correlated with bacterial CFUs ex vivo to demonstrate imaging agent sensitivity. (D) ^18^F-fluoromannitol imaging sensitivity improved approximately 20-fold using parametric imaging (left), compared with clinical standard metric of SUV (right). Arrows point to sites of histologically confirmed infection. Data are mean ± SEM. %ID = percentage injected dose; Ki = net influx rate.

We used a murine myositis model of musculoskeletal infection (10–12*,*^14^*,*^20^*,*^23^) to determine whether ^18^F-fluoromannitol can differentiate sterile inflammation from infection in vivo by inoculating the right triceps brachii with a live strain of bacteria and the left triceps brachii with a 10-times quantity of heat-killed bacteria to generate an inflammatory response. ^18^F-fluoromannitol accumulated specifically in the site of infection in both gram-positive and gram-negative strains (Fig. 3A). ^18^F-FDG was predictably unable to distinguish infection from inflammation, consistent with prior reports (10–12*,*^14^*,*^20^*,*^23^), but did serve as a valuable positive control. Dynamic imaging revealed rapid accumulation and significant differences in PET signal in as little as 5 min after ^18^F-fluoromannitol injection.

To quantify PET signal, we generated volumes of interest in the upper limbs of mice using CT for anatomic localization. ^18^F-fluoromannitol displayed a 3.5-fold increased SUV (summed frames 45–60 min after injection) compared with the contralateral site of inflammation (Supplemental Fig. 8). ^18^F-FDG could not show significant differences in SUV between sites of infection and inflammation. After the scans, we excised both triceps brachii to confirm the PET data using γ-counting, which confirmed the increased PET signal in the infected tissue compared with inflamed tissue (Supplemental Fig. 9). Biodistribution studies were performed in successive cohorts of mice over 3 h to determine the dosimetry of ^18^F-fluoromannitol (Fig. 3B; Supplemental Fig. 10). The kidneys and bladder demonstrated the highest nonspecific accumulation of ^18^F-fluoromannitol, consistent with PET imaging data. We also correlated static PET SUV with bacterial CFUs from excised tissue to determine the sensitivity of ^18^F-fluoromannitol (Fig. 3C; Supplemental Fig. 11) and found that ^18^F-fluoromannitol can reliably detect as little as 5 log_10_ (CFUs/mL) of bacteria in vivo by SUV. We also investigated whether parametric mapping (21) can increase the bacterial sensitivity of ^18^F-fluoromannitol in vivo (as net influx rate is a quantitative measure of the rate of uptake in tissue (24)), rather than SUV (which is semiquantitative and cannot delineate signal from blood pool contamination and tissue). Parametric imaging improved the bacterial sensitivity of ^18^F-fluoromannitol by roughly 20-fold (log_10_ CFUs = 1.3; 1.7 × 10^6^ improved to 7.0 × 10^5^ CFUs) (Figs. 3C and 3D; Supplemental Fig. 12).

**FIGURE 4.**
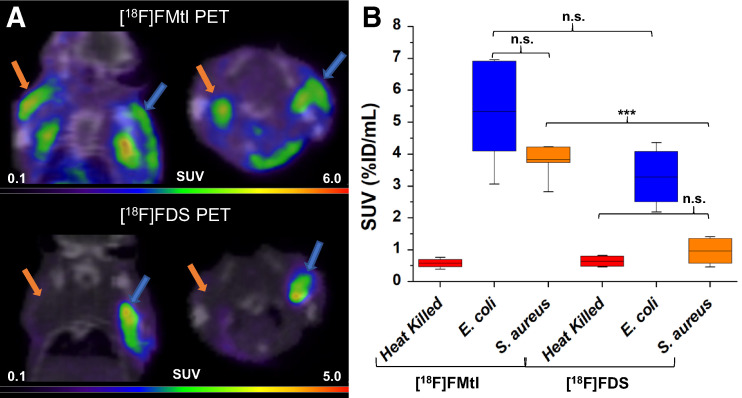
PET/CT static imaging of murine mixed myositis model of infection. (A) ^18^F-fluoromannitol PET signal was observed in both *S. aureus*–infected muscle (orange arrows) and *E. coli*–infected muscle (blue arrows). ^18^F-FDS signal is specific to *E. coli*–infected muscle and is not observed in *S. aureus* infection. (B) ^18^F-fluoromannitol and ^18^F-FDS SUV from PET scans show that both agents can detect *E. coli* with equivalent sensitivity, but ^18^F-fluoromannitol SUV is significantly higher than ^18^F-FDS SUV for *S. aureus*. ***P < 0.001. %ID = percentage injected dose; FMtl = fluoromannitol; n.s. = not statistically significant.

We also investigated the sensitivity of ^18^F-fluoromannitol compared with ^18^F-FDS in a mixed infection (polymicrobial) model. ^18^F-FDS has shown remarkable specificity for Enterobacteriaceae in vivo but has shown limited to no sensitivity toward gram-positive and other gram-negative organisms. Mice were inoculated with live *E. coli* (8.4 × 10^6^ CFUs) and *S. aureus* (8.8 × 10^6^ CFUs) in the right and left triceps brachii, respectively. No significant differences in SUV (*P* = 0.19) were observed between *E. coli* and *S. aureus* infection in the same animal (Figs. 4A and 4B) with ^18^F-fluoromannitol. Importantly, ^18^F-fluoromannitol accumulation was significantly higher than ^18^F-FDS accumulation in *S. aureus* infection (*P* < 0.001). ^18^F-FDS demonstrated high specificity for *E. coli,* compared with *S. aureus* (*P* = 0.007); however, no significant differences in *E. coli* SUV were evident between ^18^F-fluoromannitol and ^18^F-FDS (*P* = 0.11). Postmortem γ-counting of tissues confirmed ^18^F-fluoromannitol uptake in both *S. aureus* and *E. coli,* whereas ^18^F-FDS accumulated only in *E. coli*–infected muscle (Supplemental Fig. 13). In sum, our imaging data show that ^18^F-fluoromannitol accumulates in both gram-positive and gram-negative organisms and is of adequate sensitivity to serve as an in vivo broad-spectrum imaging tool for infection.

### Imaging Wound Infection with ^18^F-Fluoromannitol PET

**FIGURE 5.**
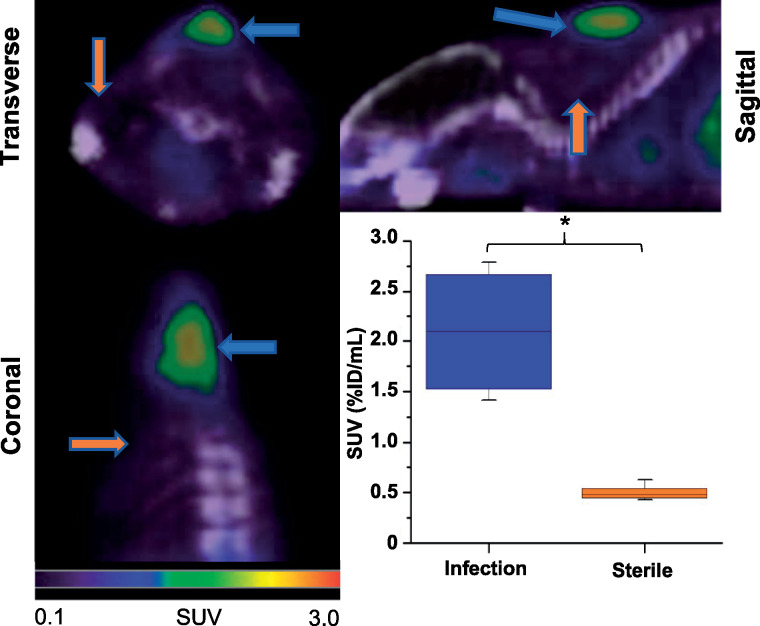
^18^F-fluoromannitol detects *A. baumannii* infection in wound of C57BL/6 mice (left). ^18^F-fluoromannitol in vivo PET imaging shows significant differences in SUV in infected wound (blue arrows), compared with sterile inflammation (orange arrows) located 1 cm caudal and sinister to infected wound (right). Data are mean and range (4 animals for each group). *P < 0.01. %ID = percentage injected dose.

The emergence of carbapenem-resistant *A. baumannii* has rendered clinical management of *A. baumannii* infections difficult to impossible (25) in some cases. The urgency to improve management of *A. baumannii* infections prompted us to investigate whether ^18^F-fluoromannitol can detect *A. baumannii* in a laceration wound model. Mice were inoculated with *A. baumannii* (9.4 × 10^6^ CFUs) through a small incision in the dorsal fascia and imaged using PET/CT. ^18^F-fluoromannitol accumulated specifically in the infected wounds of mice (Fig. 5; Supplemental Fig. 14), demonstrating nearly a 6-fold increase in SUV (7.0 log_10_ CFUs) compared with a 10-times quantity of heat-killed bacteria in a nearby wound.

### Quantifying Antimicrobial Efficacy In Vivo

**FIGURE 6.**
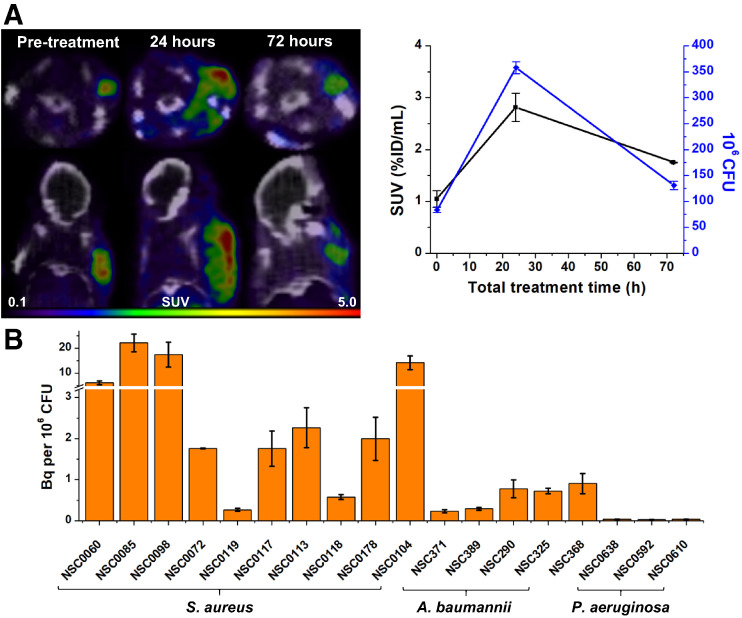
^18^F-fluoromannitol can quantify antimicrobial efficacy in vivo. (A) Mice were inoculated with clinical isolate of *S. aureus,* and antimicrobial efficacy of vancomycin was monitored over 72 h of treatment. Antimicrobial efficacy was quantified with serial PET imaging (left) and correlated with CFUs (right). Data are mean ± SEM (3 per time point). (B) Uptake of ^18^F-fluoromannitol in clinical isolates of *S. aureus, A. baumannii,* and *P. aeruginosa*. Data are mean ± SEM (6 per strain). %ID = percentage injected dose.

The growing incidence of antimicrobial resistance in many bacterial pathogens is a serious concern because treatment failure is a major threat to global health (26). Inappropriate antibiotic use is also the primary driver of antibiotic resistance (27), which also places undue risk on patients for adverse events such as allergic reactions and *Clostridium difficile* infection. Thus, it is imperative to optimize the management of infection and use of antibiotics. We investigated whether ^18^F-fluoromannitol can quantify the efficacy of antibiotic therapy in vivo. Mice were inoculated with *S. aureus* in the right triceps brachii and imaged with ^18^F-fluoromannitol 8 h after infection, before initiation of vancomycin treatment (100 mg/kg every 8 h, intraperitoneally), and subsequently were imaged at 24 and 72 h after treatment. The PET signal diminished over the course of treatment, correlating closely with CFU burden (Fig. 6A). We next investigated the accumulation of ^18^F-fluoromannitol in a panel of bacterial isolates from infected combat wounds of military service members (Fig. 6B). ^18^F-fluoromannitol demonstrated broad accumulation in *S. aureus* and *A. baumannii* but did not show appreciable accumulation in *P. aeruginosa*. Taken together, these findings indicate that ^18^F-fluoromannitol can be used as an effective tool to image a variety of clinically relevant pathogens.

## DISCUSSION

Mortality-associated infection disproportionately affects populations with strained access to health care (28); thus, a critical metric for any imaging agent is that it be easily disseminated. The use of ^18^F ensures that the isotope is regularly available from cyclotron production, and the half-life (109.5 min) facilitates widespread distribution. The radiosynthesis of ^18^F-fluoromannitol (Fig. 1) is a straightforward 3-step reaction; the first 2 steps were intentionally designed to model the radiosynthesis of ^18^F-FDG, followed by seamless sodium borohydride reduction (29). All purifications are cartridge-based and facilitate automation on any radiosynthesizer, promoting robust access to ^18^F-fluoromannitol.

Clinical management of infection typically commences with empiric antibiotic therapy using broad-spectrum agents, often combined with a targeted antimicrobial. Treatment generally continues until biopsy or culture reveals the causative organism; however, treatment may continue in lieu of positive identification. The inability to rapidly delineate bacterial infection promotes unnecessary exposure to antibiotics, contributing to the rising incidence of antimicrobial resistance mechanisms and morbidities associated with antibiotic therapy (30*,*^31^). ^18^F-fluoromannitol shows rapid and specific accumulation in bacteria in vivo in several clinically relevant pathogens. Despite the seeming disparity in accumulation of ^18^F-fluoromannitol in *E. coli* compared with ^18^F-FDS in vitro (Fig. 2D), no significant differences (*P* = 0.11) in tracer accumulation were observed in vivo in *E. coli* (Fig. 4). This important observation highlights that although in vitro assays play fundamentally important roles in preliminary characterization and validation, it is imperative that other characteristics, such as pharmacokinetics, not be overlooked when evaluating the candidacy of a novel radiopharmaceutical for imaging. Collectively, the minimal nonspecific accumulation and radioactive dose of ^18^F-fluoromannitol in mammalian tissue suggest that this agent is well poised for clinical studies on anatomic localization of a variety of infections.

Imaging can realistically play a complementary role in managing several clinical applications of infection with diverse etiologies. However, the complementary role imaging will play is ultimately limited to the in vivo sensitivity of the agent (CFUs/mL). Several radiopharmaceuticals have been studied, including glucose (^18^F-FDG) (32*,*^33^), sorbitol (^18^F-FDS) (10*,*^34^), and maltose (^18^F-fluoromaltose, ^18^F-fluoromaltotriose) (11). ^18^F-FDS has shown adequate sensitivity to infection in both preclinical models and human disease; however, this agent is limited to the detection of Enterobacterales. Maltose-derived radiopharmaceuticals demonstrated improved strain coverage that includes *P. aeruginosa* and *S. aureus*; however, the sensitivity of these agents for clinically relevant concentrations of bacteria beyond *E. coli* remains uncertain. Other imaging agents, such as those targeting folate biosynthesis (12–14) or transpeptidases (20*,*^23^), report limited (10^8^ CFUs) or unknown sensitivity. ^18^F-fluoromannitol was able to reliably detect 10^5^ CFUs in vivo using the clinical standard SUV, which is of sufficient sensitivity for detecting an abscess (3). Furthermore, the sensitivity of ^18^F-fluoromannitol did not diminish between *E. coli* and *S. aureus,* suggesting that sensitivity is not dependent on a specific genus or family of bacteria.

The Centers for Disease Control and Prevention estimates that approximately 30% of prescribed antibiotics are unnecessary (27), and it is alarming that inappropriate use of antibiotics is the primary driver for the development of antibiotic resistance mechanisms. Bacterial CFUs were shown to correlate with PET SUV during vancomycin treatment using ^18^F-fluoromannitol imaging (Fig. 6A). In addition, ^18^F-fluoromannitol demonstrates indistinguishable accumulation in *E. coli* and *S. aureus* in vivo. ^18^F-fluoromannitol is well positioned to serve as a valuable tool for diseases that are currently challenging or impossible to definitively delineate using current clinically available imaging tools, such as delineation of degenerative disk disease (sterile inflammation) from discitis osteomyelitis (infection). With the imaging tools now available, it is intriguing to envision a role in which imaging can rapidly diagnose infection (^18^F-fluoromannitol: broad spectrum) and optimize the selection of an appropriate antibiotic for the pathogen (^18^F-FDS: Enterobacterales specificity). Thus, these precision medicine tools may improve management of patient care and limit or eliminate unnecessary antibiotic use.

Our study was not without limitations. ^18^F-fluoromannitol requires active transport of mannitol mediated by the mannitol-specific phosphotransferase system in bacteria. Thus, it is possible that senescent or slow-growing bacterial populations may diminish ^18^F-fluoromannitol sensitivity. However, recent studies have shown that mannitol and fructose stimulated bacterial metabolism and enabled aminoglycoside antibiotic sensitivity (28*,*^35^*,*^36^). Further studies may be warranted to examine whether ^18^F-fluoromannitol can serve as a prognostic indicator for this type of therapeutic strategy. Our studies revealed limited accumulation of ^18^F-fluoromannitol in *P. aeruginosa,* a difficult-to-manage pathogen in patients with comorbidities (37*,*^38^). This outcome is surprising because the mannitol operon is well characterized in *P. aeruginosa* (39–41). Nonetheless, this finding is consistent with other mannitol-derived and sugar alcohol–derived radiopharmaceuticals studied (19) in *P. aeruginosa* in vitro.

## CONCLUSION

We have described a novel radiopharmaceutical, ^18^F-fluoromannitol, for imaging infections in a diverse spectrum of pathogenic organisms, including *S. aureus, A. baumannii,* and *E. coli*. Production of ^18^F-fluoromannitol is straightforward, robust, and high-yielding, thus facilitating wide accessibility. Accordingly, ^18^F-fluoromannitol might be rapidly translated to clinical studies as a noninvasive diagnostic tool facilitating rapid delineation of infection from sterile inflammatory processes, ultimately reducing the incidence of antimicrobial resistance promoted by selection pressures derived from unnecessary use of antibiotics.

## DISCLOSURE

This work is supported by NIH/NIBIB R01EB028338-01 and the Military Infectious Diseases Research Program and Defense Health Programs subcontract DoD/NMRC N3239820P0034 (Kiel Neumann). Mark Simons and Alexandria Kesterson are military service members; this work was prepared as part of their official duties. Title 17, U.S. Code, §105, provides that copyright protection under this title is not available for any work of the U.S. government. Title 17, U.S. Code, §101, defines a U.S. government work as a work prepared by a military service member or employee of the U.S. government as part of that person’s official duties. The views, opinions, or findings contained in this report are those of the authors and should not be construed as an official Department of the Navy, Department of Defense, or U.S. government position, policy, or decision unless so designated by other documentation. No other potential conflict of interest relevant to this article was reported.

## Notes

KEY POINTS **QUESTION:** What are the limitations of ^18^F-fluoromannitol imaging and availability? **PERTINENT FINDINGS:** ^18^F-fluoromannitol is produced using a simple nucleophilic substitution reaction that is deployable on virtually any commercially available synthesizer present in any nuclear pharmacy and is expected to be widely available for clinical use. ^18^F-fluoromannitol PET demonstrated high sensitivity and specificity for both gram-positive and gram-negative organisms in vivo, and PET signal was shown to closely correlate with CFU burden. **IMPLICATIONS FOR PATIENT CARE:** ^18^F-fluoromannitol signal is not limited by bacterial genus in vivo, correlates with CFU burden, and can quantify antimicrobial efficacy. ^18^F-fluoromannitol should be studied further in bacterial infections of diverse etiology in the clinical setting. The availability of this imaging tool might improve the management of deep-seated and difficult-to-manage bacterial infection.
